# Browning and Graying: Novel Transcriptional Regulators of Brown and Beige Fat Tissues and Aging

**DOI:** 10.3389/fendo.2016.00019

**Published:** 2016-03-02

**Authors:** Elisabetta Mueller

**Affiliations:** ^1^Genetics of Development and Disease Branch, National Institute of Diabetes and Digestive and Kidney Diseases, National Institutes of Health, Bethesda, MD, USA

**Keywords:** age-associated metabolic dysfunction, brown fat thermogenesis, transcription factors, HSF1, FoxA3

## Abstract

Obesity represents a major risk factor for the development of a number of metabolic disorders, including cardiovascular disease and type 2 diabetes. Since the discovery that brown and beige fat cells exist in adult humans and contribute to energy expenditure, increasing interest has been devoted to the understanding of the molecular switches turning on calorie utilization. It has been reported that the ability of thermogenic tissues to burn energy declines during aging, possibly contributing to the development of metabolic dysfunction late in life. This review will focus on the recently identified transcriptional modulators of brown and beige cells and will discuss the potential impact of some of these thermogenic factors on age-associated metabolic disorders.

## Introduction

Obesity arises when the caloric input exceeds energy output. The principal organ that expands in response to nutritional overload is white fat, a tissue endowed with the critical evolutionary role to conserve and store energy, as triglycerides, to be deployed during periods of nutrients unavailability. White adipose depots located in diverse anatomical locations, such as in the visceral cavity and under the skin, have been shown to expand differentially in response to nutritional cues, hormonal signals, and during the aging process. In antithesis to white, brown fat, mainly present in the interscapular area in rodents and rich in mitochondria interspersed among multilocular lipid droplets, is entrusted with the function of burning chemical energy to generate the heat required to maintain core body temperatures during adverse atmospheric events, such as acute or prolonged exposure to cold (1, 2). For its thermogenic function, brown fat tissue relies on UCP1, a mitochondrial protein that uncouples oxidative phosphorylation from ATP synthesis, generating mitochondrial proton leak leading to energy dissipation (3). For a number of years, it had been suspected that an additional type of fat cells existed embedded within some of the white depots and thought to be responsible for the heterogeneous, mixed histological appearance observed after changes in ambient temperatures or in response to certain pharmacological stimuli; such elusive cells have been recently isolated in mice and called beige because of their intermediate functional characteristics between white and brown adipocytes (4). Beige cells have a smooth muscle-like developmental origin (5), deriving from precursors distinct from those known to give rise to white or brown fat, possess specific gene signatures, and are contradistinguished by unique markers, such as the developmental factor Tbx1 (4, 6). In response to cold temperatures or β-adrenergic stimuli, beige cells present in subcutaneous white tissue can promptly switch from energy storage functions to programs that initiate calorie burning through the induction of a futile cycle that involves creatine metabolism (7), in addition to the activation of`UCP1-dependent thermogenic programs characteristic of brown fat cells. Because of the ability of these beige cells to mount a rapid thermogenic response, together with brown fat cells, they have been considered contributors to the overall ability of organisms to expend energy (8, 9).

## Transcriptional Modulators of Thermogenesis

The functional importance of brown fat for heat generation to protect animals from the perils of exposure to low temperatures in the wilderness has been recognized for decades and has represented the focus of studies by morphologists and biochemists for a number of years. However, only in the last 15 years, there has been an improvement in our understanding of the molecular mechanisms that guide the development and govern the function of thermogenic tissues. Recent radiographic and molecular evidence establishing the presence of brown and beige tissues also in adult humans (10–14), in addition to neonates and rodents, has sparked an interest in the possibility of identifying molecular switches to be targeted for the induction of energy consumption as anti-obesity intervention (15).

### PGC1α

One of the first advances in this field was made in the late 90s in the laboratory of Bruce Spiegelman, where Puigserver and colleagues performed a yeast two-hybrid screen to identify novel interactors of the master regulator of adipocyte differentiation, PPARγ, in brown fat tissue (16). This work led to the characterization of the peroxisome proliferator-activated receptor gamma coactivator 1α, PGC1α, as a novel coregulator of UCP1 expression, rapidly induced in brown fat upon cold exposure. It has now been recognized that PGC1α can modulate metabolism through its interaction with a number of transcription factors on promoters of genes encoding for proteins involved in metabolic functions, such as mitochondrial and peroxisomal remodeling and biogenesis, and β-oxidation in brown fat (17–19). Analysis of the *in vivo* requirements of PGC1α has demonstrated a direct involvement of PGC1α in thermogenesis and in beige fat biology as shown by the blunted response to cold challenge of mice with fat-specific deletion of PGC1α and by the decreased levels of thermogenic markers, such as UCP1, in the inguinal fat pads (iWAT) of Fat-PGC1α KO mice (20). In addition to the classic inducers of brown fat function, such as β-adrenergic stimuli and CREB signaling, known to regulate PGC1α mRNAs, it has been demonstrated that PGC1α protein levels can be regulated in an autocrine/paracrine fashion by factors such as FGF21, prompting PGC1α-dependent browning of iWAT (21). Given the role of PGC1α as a critical transcriptional coregulator of energy balance, a large emphasis has been placed on the identification of the upstream factors and signaling pathways that activate PGC1α and on the characterization of PGC1α downstream targets to ultimately enhance energy expenditure.

### PRDM16

To further understand the mechanisms regulating brown fat physiology and to identify the key contributors to brown fat identity, a few years after the discovery of PGC1α, the Spiegelman laboratory carried out a systematic search for transcriptional regulators differentially expressed in brown fat tissue in comparison to epididymal white depots. Through this analysis, Seale and colleagues identified the zinc finger protein PR domain containing 16, PRDM16, as a brown fat selective cofactor able to activate brown fat gene programs (22). Mechanistically, it was demonstrated that PRDM16 can modulate UCP1 expression *via* its direct interaction with PGC1α and β. Studies of putative brown fat depots of WT and PRDM16 global knockout mice at embryonic day 17 showed reduced expression of thermogenic genes and elevation of muscle-specific genes supporting a role for PRDM16 as an early determinant of brown fat lineage and as a negative regulator of muscle development (23). Analysis of transgenic mice with conditional expression of PRDM16 in white fat driven by the promoter of the fatty acid binding protein, aP2, demonstrated that PRDM16 is also involved in the development of beige adipocytes in subcutaneous fat and that it induces thermogenic genes, such as Ucp1, Cidea, Cox8b, and Elovl3, in these cells. The molecular changes induced by *in vivo* overexpression of PRDM16 in fat tissue were associated with increased whole body energy expenditure and protection from the weight gain induced by high-fat diet, indicating a selective role for PRDM16 in adaptive thermogenic responses mediated by beige cells (24). Conversely, analysis of mice with ablation of PRDM16 selectively in adipose tissues demonstrated that the absence of PRDM16 is associated with a switch in the molecular and morphological characteristics of inguinal fat into those of epididymal WAT. Mice with fat selective ablation of PRDM16 exposed to high-fat and -carbohydrate diet for 16 weeks maintained at room temperature developed obesity and insulin resistance (25). Of note, the effects of PRDM16 ablation in fat tissues driven by the adiponectin promoter appeared to be inguinal fat specific, causing the depletion in beige cells but not altering brown fat tissue functionality. Overall these gain- and loss-of-function studies performed both *in vitro* and *in vivo* have provided evidence for a role of PRDM16 in the regulation of brown and beige fat tissues maintenance and in restricting muscle developmental programs.

### PRDM3

Recently, Harms and colleagues in the laboratory of Seale demonstrated that PRDM3, a factor closely related to PRDM16, plays a role in establishing brown fat identity (26). PRDM3 induces UCP1 and PGC1α when overexpressed in C2C12 cells. PRDM3 levels appear to be highly regulated and are shown to decline in brown fat tissue as the mice age, with the highest levels observed at embryonic stage 18. These data suggest that PRDM3 may complement the function of PRDM16 during early developmental phases. This possibility is supported by the evidence that double knockout mice for both PRDM3 and PRDM16 have marked decrease in brown fat formation. Recent molecular data have demonstrated that, similarly to PRDM16, PRDM3 can alter chromatin structure at brown fat gene-specific promoters *via* its interaction with MED1, thereby enhancing target gene expression (27). Given that PRDM3 has been reported to play broad fundamental functions in heterochromatin maintenance (28), future studies may reveal how PRDM3 regulates gene transcription in a depot-selective manner and whether it may rely on additional – yet to be identified – tissue-specific interacting partners to achieve brown fat tissue effects.

### TAF7L

Another important molecular determinant of brown fat was recently discovered in the laboratory of Robert Tijan. Zhou and colleagues reported that the TATA-binding protein-associated factor 7L (TAF7L), previously shown to be a critical regulator of white adipose tissue differentiation, also functions as a molecular switch between brown fat and muscle lineages (29). Through studies involving histological and molecular analysis, Zhou and collaborators showed that TAF7LKO mice have decreased brown fat tissue and increased muscle mass. These effects are associated with upregulation of genes involved in skeletal muscle development and function, and a decrease in brown fat gene expression programs. Gain- and loss-of-TAF7L-function studies performed in cell lines, such as 10T1/2 and C2C12, further demonstrated that TAF7L modulates mesenchymal cells fate. To determine the mechanisms through which TAFL4 may tip the balance toward the brown adipose lineage, the authors performed immune-precipitation studies and identified a fat-specific complex containing only a subset of canonical TFIID-TAF subunits co-purifying with TAF and PPARγ. These TAF7L-containing TFIID complexes appear to mediate DNA looping between distal enhancers and core promoter elements in adipose tissues, suggesting that TAF7L is a tissue-specific subunit of TFIID involved in the coordination of long-range chromatin interactions to specify brown fat lineage. These studies suggest the possibility of the existence of novel additional fat depot-selective TFIID complexes each involved in the determination of a distinct fat cell type.

### Zfp516

In search for novel regulators of thermogenic tissue function, Dempersmier and colleagues in the group of Hei Sook Sul performed a high-throughput screen of factors binding to the UCP1 promoter. Through this analysis, it was shown that the Kruppel-like zinc finger protein Zfp516 directly binds to a proximal region of the UCP1 promoter present at −70 to −45 bp from the start site (30). More detailed molecular analysis revealed that Zfp516 is induced upon cold exposure and that it regulates UCP1 gene expression in complex with the coactivator PRDM16. Analysis of promoter sequences upstream of a number of brown fat genes revealed the presence of sequence similarities with the CCACT DNA stretch identified in the UCP1 promoter, and bound by Zfp516, and ChiP studies confirmed the ability of Zfp516 to occupy a similar motif also in the promoter of PGC1α and Cox8b. Analysis of inguinal fat depots of mice with overexpression of Zfp516 driven by the −5.4 aP2 promoter kept at room temperatures showed increased clusters of cells containing multilocular lipid droplets, elevated UCP1 staining, consistent with increased amounts of beige fat cells. Furthermore, it was shown that these mutant mice have higher oxygen consumption levels. Assessment of the specific tissues contributing to increased respiratory activity in mice overexpressing Zfp516 revealed a selective effect of Zfp516 in inguinal fat, while no changes in respiration were observed in brown fat tissue. Consistent with the effects resulting from increased number of beige fat cells in iWAT, Zfp516 transgenic mice showed elevation in their core basal temperatures, had improved cold tolerance, and were protected from diet induced obesity. Global ablation of Zfp516, although lethal, allowed the analysis of the effects of ablation of Zfp516 on the early developmental phases of brown fat formation. Embryos lacking Zfp516 at day 20.5 showed reduced brown fat mass and decreased brown fat gene expression in presumptive BAT. Overall, the data obtained in the two genetic models described highlight a dual role for Zfp516 in controlling the development of brown tissue and also in the modulation of beige fat cells function in adult mice.

### P107

To characterize novel transcriptional regulators involved in the commitment of stem cells toward the adipocyte lineage, Scimè and colleagues (31) investigated the role of the Rb family member p107, previously shown to modulate white adipocyte differentiation through its repression of PGC1α (32). The expression of p107 appeared to be tightly controlled given that it was found to be expressed selectively in white fat stem cells but completely absent from those giving rise to brown fat. Loss-of-function studies demonstrated that p107 ablation is permissive for the formation of brown fat adipocytes and required for PRDM16-mediated brown fat programing of mesenchymal stem cells. Additional studies will determine the specific mechanisms through which this transcriptional corepressor ultimately influences brown versus white fate choices.

### Ewing Sarcoma

The group of Sean Lee recently demonstrated an unexpected, novel role for the Ewing Sarcoma (EWS) factor in brown fat biology (33). Park and colleagues showed that in the absence of EWS, pups die within 24 h after their birth and that the only pathological abnormality they manifest is a marked reduction in brown fat tissue. Histological analysis of the presumptive BAT present in these embryos revealed reduced UCP1 staining, loss of the characteristic multilocular lipid droplet phenotype and molecular studies demonstrated decreased levels of factors such as PGC1α and PRDM16. Analysis of brown adipocyte differentiation in immortalized preadipocytes generated from EWS WT and null newborn pups showed that EWS null cells are unable to differentiate when treated with a differentiation-inducing cocktail. To gain further clues to mechanistically define how EWS affects differentiation, the authors studied the patterns of gene expression in WT and EWS null cells and observed that EWS deficiency affected the mRNA levels of the gene encoding for the bone morphogenic protein 7 (BMP7), as well as for CEBPβ during the initial stages of differentiation. Specific analysis of the effects of EWS on the expression of this cell fate determination factor revealed that EWS occupies the BMP7 promoter when in a complex with YBX1, a multifunctional protein involved in both transcription and translation. Complementation studies employing exogenous BMP7 restored differentiation in EWS null cells demonstrating that lack of BMP7 constituted their differentiation block. To assess whether lack of EWS would affect beige cells function, the authors studied EWS heterozygous mice and showed that they have significantly reduced beige gene activation in response to rosiglitazone and β-adrenergic stimulation. These studies demonstrate novel tissue-specific functions of EWS as a brown fat determination factor through the control of the early cell fate steps involving the activation of BMP7 and suggest a possible role in EWS in the activation of beige cells in response to pharmacological treatment. Studies of fat tissue conditional animal models will permit the assessment of the contribution of EWS on mature brown fat functionality.

### Foxa3

To specifically identify novel transcriptional regulators involved in early events regulating fat differentiation, Xu and colleagues performed a genetic screen using a si-RNA library and assessed the effects of knocking down the expression of each forkhead protein on adipogenesis (34). This systematic analysis led to the demonstration that the winged helix factor Foxa3 is a novel modulator of adipocyte differentiation and of its function. Analysis of WT and Foxa3 null mice revealed that the absence of Foxa3 *in vivo* can decrease the expansion of the visceral adipose tissue compartment in response to HFD but does not reduce the enlargement of the subcutaneous fat tissue. This depot-selective protection from the development of intra-abdominal obesity during high-fat diet regimens (34) was associated with improved insulin sensitivity. Analysis of the long-term effects of Foxa3 deficiency on late onset metabolic dysfunction revealed that mice lacking Foxa3 kept on a normal chow diet for more than a year have increased browning of subcutaneous tissue, as indicated by increased UCP1 staining in iWAT and elevated expression of a number of classic thermogenic and lipid oxidation genes in addition to beige specific markers such as Tbx1. These morphological and gene expression changes in iWAT, consistent with the acquisition of a beige phenotype, were associated with increased oxygen consumption (35). Analysis of Foxa3’s mode of action indicated that Foxa3 controls calorie hoarding through the upregulation of PPARγ levels *via* transcriptional cooperation with CEBPs, as revealed by coimmunoprecipitation and ChIP studies demonstrating that CEBPs interact with Foxa3 at the PPARγ promoter. In addition, Foxa3 suppresses thermogenesis in BAT and iWAT through interference with CREB-mediated induction of PGC1α expression. Analysis of the changes in Foxa3 mRNA levels in response to HFD and during the course of aging revealed that Foxa3 is fat depot-selectively induced in response to nutritional and developmental cues. Specifically, while diets rich in fat lead to increased Foxa3 mRNA levels only in the visceral fat depot, and not in subcutaneous adipose tissue, the aging process is associated with increased Foxa3 levels most prominently in subcutaneous fat and in BAT. Given the role that other Foxa family members have been shown to play as pioneering factors on chromatin remodeling (36), it is plausible that Foxa3 may exert its calorie hoarding function through a number of other mechanisms in addition to those involving the control of PPARγ and PGC1α and their downstream programs. Ongoing studies in the laboratory are characterizing novel Foxa3’s gene targets in distinct fat subtypes and unraveling the signals regulating the unique pattern of expression of Foxa3 in selected depots.

### HSF1

To identify novel modulators of fat tissue browning, Ma and colleagues used PGC1α expression as a read-out and performed an *in silico* screen of possible functional binding sites located upstream of the PGC1α promoter (37). This analysis led to the identification of a putative heat shock motif (HSE) and studies involving overexpression of HSF1, or HSF1 activation *via* its agonists, in isolated cells and *in vivo*, demonstrated that HSF1 induces PGC1α and that it can turn on a cascade of mitochondrial PGC1α-dependent gene programs. ChIP assays revealed that upon cold exposure, HSF1 occupies the heat shock element present in the PGC1α promoter in both BAT and iWAT, and coimmunoprecipitation studies demonstrated that HSF1 physically interacts and functionally cooperates with PGC1α in a feed-forward regulatory loop on the PGC1α promoter. Given the *in vitro* evidence of HSF1 involvement in PGC1α activation, it was hypothesized that absence of HSF1 *in vivo* would bring about abnormal metabolic functions. Analysis of the effects of ablation of HSF1 on a number of parameters demonstrated that HSF1 KO mice are more sensitive to low temperatures. Furthermore, morphological and molecular analysis of adipose tissues of WT and HSF1 KO mice revealed increased lipid deposition in BAT and iWAT of mice lacking HSF1, reduced UCP1 staining, decreased UCP1 protein levels in inguinal fat tissues, and reduced thermogenic and β-oxidation expression programs, indicating overall reduced brown and beige tissues functionality. Genetic experiments involving gain-of-HSF1-function selectively in iWAT and in muscle enabled through adenoviral-mediated expression of HSF1 provided further confirmatory evidence that HSF1 increases mitochondrial and thermogenic gene programs and leads to elevation in energy consumption. Analysis of the consequences of pharmacological activation of HSF1 during high-fat diet *via* celastrol, a natural HSF1 agonist present in herbal extracts used in Chinese medicine, revealed enhanced subcutaneous fat browning, increased thermogenesis and energy consumption, and reduced adipose tissue expansion, providing a proof of concept that HSF1 may represent a possible target in obesity prevention. Of note, it has been recently reported that celastrol is also efficacious in inducing weight loss in obese mice through a mechanisms involving appetite suppression *via* leptin sensitization (38). Although it appears that celastrol prevents and treats obesity through distinct pathways, the evidence that obese mice treated with celastrol lose weight even when their food intake is equalized to that of controls suggest that celastrol may activate the HSF1 pathway and increase energy expenditure also in obese states. Ongoing studies are dissecting genetically the contribution of distinct organs to HSF1-mediated modulation of energy expenditure and discriminating between HSF1 effects on central regulatory signals and those on peripheral organs.

### KLF11

Loft and colleagues in the Mandrup laboratory have recently demonstrated a novel role for the Kruppel-like factor, KLF11, in the control of browning of human adipocytes (39). KLF11, also known as Mody 1, had been previously shown to regulate pancreatic β-cell function and variants of this gene have been shown to be associated with diabetes. The Mandrup group has now provided novel evidence that KLF11 is highly expressed in beige cells and that it is a direct target of agonist liganded PPARγ. This factor induces reprograming of beige cells in response to TZDs through functional cooperation with PPARγ and selective activation of brown fat super-enhancers, suggesting a novel role for KLF11 in the stabilization of the expression of beige gene targets in human cells.

### IRF4

Kong and colleagues in the Rosen group recently characterized the interferon regulatory factor 4 (IRF4) as a transcriptional regulator of adaptive thermogenesis (40). IRF4 had been previously studied in the context of white adipocytes and shown to have an anti-lipogenic activity through the control of genes such as adipocyte triglyceride lipase and hormone-sensitive lipase (41). In the new analysis, Kong and colleagues show that IRF4 is induced by cold exposure in brown fat cells. This pattern of expression prompted the assessment of the function of IRF4 also in BAT, in addition to that previously described in white fat. Analysis of brown fat-specific IRF4 transgenic mice demonstrated that IRF4 is sufficient to increase energy expenditure and to protect from diet-induced obesity. IRF4 overexpressing mice show smaller brown fat size, reduced lipid stores, and increased expression of a selected number of genes involved in thermogenesis. Of note, IRF4 overexpression induced browning in epididymal WAT but not in iWAT. Conversely, selective ablation of IRF4 driven by the UCP1 promoter was associated with reduced energy expenditure, increased predisposition to diet induced obesity and cold intolerance. Interestingly, in contrast to the selective “beigeing” effects in epididymal WAT of IRF4 overexpressing mice, UCP1 promoter-driven ablation of IRF4 reduced beige cells in iWAT of mice chronically exposed to low temperatures. Mechanistically, IRF4 cooperates with PGC1α, *via* reciprocal gene expression regulation, direct protein–protein interaction and transcriptional cooperation in the induction of thermogenic genes. These data suggest that IRF4 coordinates heat generation by modulating lipolysis in white fat and thermogenic gene expression programs in brown and beige fat depots.

### Zic 1

It has been recently suggested that Zic 1, a member of a family of zinc finger factors of the cerebellum previously involved in a variety of developmental processes, may also play a role in brown fat development and function. Zic 1 involvement in BAT biology was initially suggested when it was identified as one of the top genes differentially expressed in brown fat in comparison to white fat (22). A number of studies have now provided evidence that Zic 1 expression is restricted to brown fat cells, even in undifferentiated states, and that its levels are independent of changes in temperatures and dietary states, proposing Zic 1 as a selective marker of brown adipose tissue. *In vitro* analyses have only recently started to address the function of this transcriptional regulator. Studies performed by Nedergaard’s group have demonstrated that Zic 1 downregulation *via* knockdown is associated with blunted UCP1 induction in response to norepinephrine stimulation (42). Additional functional and physiological studies possibly involving genetic mouse models with conditional modulation of Zic 1 levels in brown fat will define more precisely whether Zic 1 is strictly required for BAT development or for its functionality in adulthood or for both.

### Zbtb16

To identify novel regulators of adaptive thermogenesis, Plaisier and colleagues analyzed transcriptomes coordinately regulated upon cold exposure in both BAT and muscle and identified Zbtb16 among the most robustly expressed genes in these conditions (43). Zbtb16 (also called PLZF) had previously emerged from a genome-wide screen of genes modulating adipocyte differentiation (44) and shown to suppress differentiation in L1 cells; however, no studies linked Zbtb16 to BAT function. In the studies by Plaisier and colleagues, overexpression of Zbtb16 was associated with increased expression of brown fat makers, such as UCP1, PGC1α, PPARα, and mitochondrial and β-oxidation genes. Detailed analysis of mitochondrial energetics revealed increased respiration in cells, such as primary adipocytes and C2C12 myoblasts, with overexpression of Zbtb16 and showed increased mitochondrial biogenesis. Survey of the expression of Zbtb16 in tissues such as white fat and heart of 100 different mouse strains indicated that Zbtb16 levels inversely correlate to metabolic traits, such as increased body weight and fat mass. The generation and the detailed characterization of conditional genetic models of this factor will aid in the definition of the physiological function of Zbtb16 in brown and/or beige fat physiology.

## Thermogenesis and Aging

It has been reported that the aging process is associated with a selective expansion and redistribution of white fat stores specifically in the visceral compartment and with progressive metabolic decline (45). Evidence indicates that not only the amount of thermogenic fat tissues decreases during the aging process due to apoptosis but that its thermogenic functionality is reduced in old animals and in aged humans due, at least in part, to decreased sensitivity to β-adrenergic stimulation (46). Specifically in mice, it has been observed that as they approach mid age, their brown adipose tissue gradually involutes. It has been hypothesized that the progressive atrophy observed may be endogenously controlled and determined by the decrease in gonadal hormones, known to support the viability and functionality of BAT in maturity, and by the tight negative control exerted by cortisol (46). Given the impact of brown fat and beige cells on energy balance, preservation, and/or restoration of functional brown and beige tissues may be an attractive strategy to protect from obesity and metabolic disorders that arise at an old age (47); however, to date, the main transcriptional switches that slow down, or accelerate, the involution of this tissue during late life stages have not been identified. Although the majority of the factors reviewed here have been shown to affect either the development of thermogenic tissues or their function in adult mice, only a few of them have been fully characterized in mid age or older mice. Specifically, it has been analyzed the long-term impact of the ablation PRDM16 specifically in the brown adipose lineage (Myf5-ΔPRDM16) (26). These studies revealed that 11-month-old Myf-ΔPRDM16 KO mice have increased whitening of brown fat tissue and reduced oxygen consumption levels; however, despite their decreased energy output and dysfunctional BAT, they do not show increased predisposition to obesity compared to age-matched WT controls. It remains to be determined whether the absence of an obese phenotype in these mice is animal model-dependent or whether compensatory mechanisms counteract the accretion of white fat stores in these mice. Indeed, given that PRDM3 offsets the effects of PRDM16 ablation during early development, it is conceivable that other factors, or other PRDM family members, may partly complement and compensate for the loss of PRDM16 late in life. Alternatively, it is possible that obesity would become evident only at later stages in life due to small, cumulative effects on fat stores. Nonetheless, metabolic studies of Myf-ΔPRDM16 mice older than 1 year or of old PRDM16 fat KO mice with impoverished beige cell contingent and measurement of longevity in diverse PRDM16 KO and transgenic mice will better define the role of PRDM16 in age-associated metabolic dysfunction and lifespan extension.

**Figure 1:**
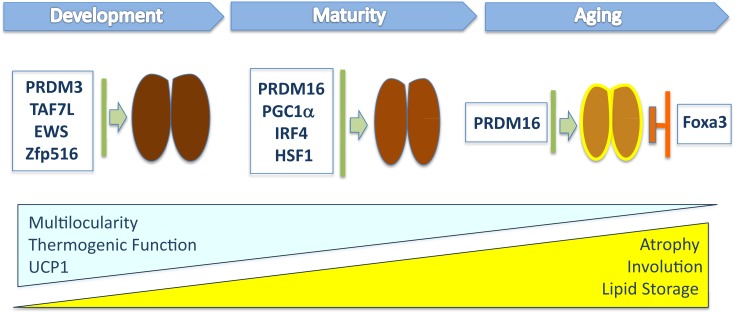
**This illustration depicts the transcriptional regulators that have been shown to modulate *in vivo* brown fat tissue during organogenesis, maturation, and involution**. The blue triangle indicates the progressive loss of multilocular lipid content, thermogenic function, and UCP1 levels during aging, while the yellow one indicates the increased whitening of BAT, composed of cells containing large lipid deposits, and atrophy observed in old mice.

Analysis of the metabolic profiles of WT and Foxa3 null mice have provided supportive evidence that increasing beige fat cells in aging may be beneficial in preventing metabolic dysfunction and even extending life. *In vivo* ablation of Foxa3 was shown to be associated with a lean phenotype, increased energy expenditure, and improved insulin sensitivity in mice older than 1 year fed a normal chow diet (35). The metabolic beneficial effects of Foxa3 ablation appeared to be associated with increased browning of iWAT in 14-month-old mice. In addition, it was observed that ablation of Foxa3 positively impacted longevity contributing to lifespan extension. Analysis of gene expression revealed that Foxa3 levels are robustly upregulated selectively in inguinal and brown fat depots in 14-month-old mice compared to levels observed in young 2-month-old mice. Overall, the analysis of the consequences of Foxa3 ablation in late life stages suggests that Foxa3 may constitute a potential target to counteract aging and its associated metabolic pathologies (Figure 1). These studies support the idea that systematic metabolic profiling of knockout or transgenic mice with altered expression of transcriptional regulators of brown and beige fat during the aging process will permit a more detailed mechanistic understanding of the impact of thermogenic tissues on metabolic disorders that occur late in life.

It has been demonstrated that brown and beige thermogenic cells increase energy expenditure and suggested that turning on these cells to boost consumption may represent a successful approach to combat the rampant obesity epidemic (8, 15). As we improve our understanding of the key molecular switches that can modulate thermogenesis, a number of questions remain to be answered to fully exploit the therapeutic potential of these cells in counteracting metabolic dysfunction at different life stages. For example, it needs to be clarified how changes in hormonal signals occurring during the aging process can contribute to the progressive involution of brown fat tissue. Given some of the reports suggesting that cortisol levels increase during aging (48), assessing how steroid hormones may regulate the expression of thermogenic effectors late in life could provide clues on some of the mechanisms through which endogenous signals unable or disable the functionality of brown and beige cells. Furthermore, the generation of new animal models to allow the modulation of the expression of specific transcriptional regulators in a spatiotemporal-restricted manner – fat depot-selectively at an old age – will be beneficial in determining which thermogenic factors may be targeted to prevent the functional decay of brown and beige fat cells shown to occur during the aging process.

## Author Contributions

The author confirms being the sole contributor of this work and approved it for publication.

## Conflict of Interest Statement

The author declares that the research was conducted in the absence of any commercial or financial relationships that could be construed as a potential conflict of interest.
